# Notching up neural stem cell homogeneity in homeostasis and disease

**DOI:** 10.3389/fnins.2014.00032

**Published:** 2014-02-25

**Authors:** Claudio Giachino, Verdon Taylor

**Affiliations:** Department of Biomedicine, University of BaselBasel, Switzerland

**Keywords:** Notch signaling, subventricular zone, hippocampal dentate gyrus, neurogenesis, aging

## Abstract

Adult neural stem cells (NSCs) are perceived as a homogeneous population of cells that divide infrequently and are capable of multi-lineage differentiation. However, recent data revealed that independent stem cell lineages act in parallel to maintain neurogenesis and provide a cellular source for tissue repair. In addition, even within the same lineage, the stem and progenitor cells are strikingly heterogeneous including NSCs that are dormant or mitotically active. We will discuss these different NSC populations and activity states with relation to their role in neurogenesis and regeneration but also how these different stem cells respond to aging. NSCs depend on Notch signaling for their maintenance. While Notch-dependence is a common feature among NSC populations, we will discuss how differences in Notch signaling might contribute to adult NSC heterogeneity. Understanding the fate of multiple NSC populations with distinct functions has implications for the mechanisms of aging and regeneration.

## Introduction

Neural stem cells (NSCs) remain neurogenic during postnatal life within two regions of the mammalian brain, the subgranular zone (SGZ) of the hippocampal dentate gyrus (DG) and the subventricular zone (SVZ) of the wall of the lateral ventricles. The discovery of adult neurogenesis in mammals opened new avenues for research in regenerative medicine, with a goal of manipulating endogenous NSCs for brain repair (Gage and Temple, 2013). Over the last twenty years we have made considerable progress in our understanding of adult neurogenesis in homeostasis and disease (Ming and Song, 2011). However, several gaps remain in our knowledge that have to be filled before the potential of adult NSCs for regeneration can be fully exploited.

One such gap, and a field of active research, relates to the presence of heterogeneous subpopulations of NSCs within the same neurogenic niche (Alvarez-Buylla et al., 2008; Bonaguidi et al., 2012). The properties, functions, and lineage relationships between these subpopulations are still ill defined. Notch signaling is one molecular pathway involved in the regulation of NSC maintenance, proliferation, quiescence, and cell fate decisions. We propose that Notch signaling strength and diversity of downstream targets might, in concert with other molecular pathways, niche signals, and intrinsic factors, contribute to establishing adult NSC heterogeneity.

## The adult neurogenic lineage in short

Work from the 90's and early 2000's discovered the sequential developmental stages that cells undergo during lineage progression within the adult neurogenic niches (Alvarez-Buylla et al., 2001; Kempermann et al., 2004). Adult NSCs share ultrastructural and antigenic features with astrocytes (Kriegstein and Alvarez-Buylla, 2009). Most adult NSCs are quiescent or intermittently dividing, which explains their resistance to antimitotic drug and high dose tritiated-thymidine treatment (Morshead et al., 1994; Doetsch et al., 1999; Seri et al., 2001). In the SVZ, NSCs that proliferate likely undergo asymmetric cell divisions to self-renew and generate committed intermediate progenitors (IPs). These mitotically active IPs generate neuroblasts that migrate to the olfactory bulb (OB) where they mature into multiple types of neurons (Alvarez-Buylla et al., 2008). In the SGZ, NSCs also generate IPs that pass through a series of maturation stages before they differentiate into neuroblasts and subsequently granule neurons of the DG. This early work already implied some degree of heterogeneity in adult NSC and IP populations (Kempermann et al., 2004), suggesting that progression through the neurogenic lineage may be a more complex process than the simple NSC to IP to neuroblast transition. Later work added new insights into this emerging complexity and even proposed the coexistence of independent stem cell lineages acting in parallel.

## Adult neural stem and progenitor cell heterogeneity within the same lineage

The ability of adult NSCs to survive antimitotic drug treatment and regenerate the neurogenic lineage suggested that they rarely divide (Doetsch et al., 1999; Seri et al., 2001). This relative quiescence of NSCs is potentially linked to their potential for long-term maintenance over the lifespan of an organism (Kippin et al., 2005; Porlan et al., 2013). Indeed, quiescence of adult NSCs is actively promoted through several mechanisms including BMP and Notch signaling (Imayoshi et al., 2010; Mira et al., 2010), neurotransmitters (Berg et al., 2013), cell adhesion molecules (Kokovay et al., 2012), cell-cycle inhibitors (Kippin et al., 2005), and intrinsic factors (Martynoga et al., 2013). However, quiescence is not a prerequisite for somatic stem cell identity, and proliferating stem cell exist alongside more quiescent stem cell pools in several organs (Li and Clevers, 2010). A similar situation of dual stem cell populations may also exist in the neurogenic niches of the adult mammalian brain. A small but detectable fraction of adult NSCs proliferate quite rapidly in both the SVZ and SGZ (Encinas et al., 2011; Ponti et al., 2013). Even though NSCs can shuttle between active and quiescent states (Lugert et al., 2010; Bonaguidi et al., 2011), not all NSCs that proliferate re-enter a quiescent state, but some divide repeatedly (Lugert et al., 2010; Encinas et al., 2011; Ponti et al., 2013). Similarly, although quiescent NSCs enter cell-cycle to generate active NSCs (Bonaguidi et al., 2011; Encinas et al., 2011) and their exit from the quiescent state is augmented in response to neurogenic stimuli and during regeneration (Morshead et al., 1994; Doetsch et al., 1999; Lugert et al., 2010; Lopez-Juarez et al., 2013), it remains unclear what proportion of NSCs can be activated during homeostatic neurogenesis. Thus, it is possible that active and quiescent NSCs represent distinct pools where transition between the two is limited and tightly controlled, at least in the absence of injury, rather than all adult NSCs being equal in their ability to switch from quiescence to activity and back.

Discrimination of quiescent from activated NSC populations and niche astrocytes has proven difficult, but progress has been made toward this end in recent years (Pastrana et al., 2009; Beckervordersandforth et al., 2010; Mira et al., 2010; Ferron et al., 2011). Active NSCs within the SVZ express the epidermal growth factor receptor, respond to EGF with increased proliferation, and are eliminated by antimitotic drug treatment, similar to IPs, but retain expression of astrocytic and radial glia markers (Pastrana et al., 2009; Giachino et al., 2014). In the adult DG, a non-radial morphology correlates with the active state (Suh et al., 2007; Lugert et al., 2010), but molecular markers that demarcate quiescent and active NSC populations have still to be defined. Interestingly, genetic lineage tracing using promoters that potentially over-represent either quiescent or active cells suggest differences in the contribution of these populations to long-term neurogenesis (DeCarolis et al., 2013). Identifying markers that discriminate between NSCs with distinct proliferative activities is needed to develop genetic tools for independent and simultaneous lineage tracing of active and quiescent NSC pools, and determine their respective lifespan and inter-conversion *in vivo*. Lineage tracing approaches with inducible-cre/flp technologies and intersectional analyses combining promoters of cell-cycle marker genes with NSC marker genes may be useful to this end.

Heterogeneity in the adult neurogenic lineage is not restricted to NSCs. In the SVZ, a proportion of Ascl1+ IPs expresses radial glia markers whereas others do not (Pastrana et al., 2009; Giachino et al., 2014). In the SGZ, a combination of Nestin, Ascl1, Tbr2, and Doublecortin expression defines at least three stages of committed progenitors (Kempermann et al., 2004; Hodge et al., 2008). It is still unclear if marker heterogeneity of IPs reflects differences in their self-renewal abilities, but it is interesting that differences in cell-cycle dynamics of IP populations in the SVZ have been recently reported (Ponti et al., 2013). Moreover, late IPs and neuroblasts make up a large fraction of proliferating cells in both neurogenic niches (Hodge et al., 2008; Lugert et al., 2012; Ponti et al., 2013), proposing that substantial lineage amplification occurs right before cell-cycle exit and terminal differentiation.

## Heterogeneous NSC lineages acting in parallel

Adult NSCs are capable of multi-lineage differentiation and able to clonally generate multiple neural lineages under appropriate conditions *in vitro* (Suh et al., 2007; Pastrana et al., 2011). Moreover, they show some degree of lineage plasticity upon heterotopic or heterochronic transplantation into neurogenic niches distinct to their origin (Suhonen et al., 1996; Sequerra et al., 2010). Indeed, elegant lineage tracing experiments demonstrated that DG NSCs are bi-potent at the single cell level and self-renew *in vivo* (Suh et al., 2007; Bonaguidi et al., 2011). SVZ NSCs are able to generate neurons, astrocytes and oligodendrocytes at the population level *in vivo* (Alvarez-Buylla et al., 2001; Menn et al., 2006; Benner et al., 2013). However, and apparently in contrast to these findings, other fate-mapping and transplantation studies demonstrated that SVZ NSCs are heterogeneous and their spatial distribution correlates with cell fate (Kelsch et al., 2007; Merkle et al., 2007). NSCs show a preference toward the production of distinct types of neurons depending on their dorsal-ventral position within the adult SVZ, and this differentiation bias seems to be intrinsic as it is maintained after *in vitro* passage and heterotopic transplantation (Merkle et al., 2007). In the same line, NSCs cultured in the absence of growth factors are able to generate either neurons or oligodendrocytes, in addition to astrocytes, but not both neurons and oligodendrocytes within the same lineage tree (Ortega et al., 2013). NSCs with oligodendrocytic potential are enriched in the dorsal and anterior SVZ (Gritti et al., 2002; Ortega et al., 2013). Genetic fate mapping indicates that these heterogeneous NSC populations in the adult SVZ are remnants of distinct germinative niches of the embryonic forebrain and derive from Emx1, Dbx1, Gsh2, Nkx2.1 or Nkx6.2 expressing lineages (Kohwi et al., 2007; Ventura and Goldman, 2007; Young et al., 2007; Lopez-Juarez et al., 2013; Merkle et al., 2014). These data suggest that the SVZ is arranged as a mosaic that is established during embryogenesis and the location within the niche can predict the type of progeny that NSCs give rise to. Whether this heterogeneity of lineages is intrinsically determined or regulated by extrinsic cues at distinct locations within the niche, or a combination of both, is still a matter of debate (Sequerra et al., 2013). The fact that manipulation of morphogen signals skews NSC differentiation potential *in vivo* suggest that the local environment plays a role in regional specification of adult NSCs (Ihrie et al., 2011). Moreover, pathological conditions stimulate NSCs to produce progenies that are different from the ones generated during homeostasis and these can migrate toward non-canonical locations, suggesting that cell fate is not fixed (Kernie and Parent, 2010; Ohira, 2011). Defining the degree of lineage plasticity of adult NSCs and the signals that can override their intrinsic programming has important implications for developing cell replacement strategies based on the mobilization of endogenous cells.

## Contribution of Notch signaling to heterogeneity of the adult NSC pool

Notch signaling is a key mediator of NSC maintenance in the developing and adult brains (Ables et al., 2011; Pierfelice et al., 2011). Notchs are transmembrane receptors and undergo a series of proteolytic cleavages that liberate the Notch intracellular domain (NICD) upon binding to their ligands. The canonical Notch signal links the NICD to the nuclear CSL (RBP-J in mice) transcriptional complex (Mumm and Kopan, 2000). The activity of Notch target genes of the *Hes*/*Hey* family is fundamental for maintaining NSCs in an undifferentiated state, suppressing the expression of proneural genes including Ascl1 (Louvi and Artavanis-Tsakonas, 2006). Inhibition of Notch or RBP-J in adult NSCs results in NSC loss and impaired neurogenesis (Breunig et al., 2007; Andreu-Agullo et al., 2009; Ables et al., 2010; Aguirre et al., 2010; Chapouton et al., 2010; Ehm et al., 2010; Imayoshi et al., 2010; Lugert et al., 2010; Imayoshi and Kageyama, 2011). Canonical Notch signaling activity and *Hes5* expression in particular distinguish NSCs from IPs in the developing and adult brain (Basak and Taylor, 2007; Mizutani et al., 2007; Andreu-Agullo et al., 2009; Imayoshi et al., 2010; Lugert et al., 2010; Giachino et al., 2014). It is unclear if Notch signaling contributes to adult NSC heterogeneity, but studies revealing that impairing distinct Notch pathway components affects distinct stages of the neurogenic lineage suggest that this might be the case. Notch1 promotes adult NSC proliferation whilst maintaining the undifferentiated state, proposing a role of the pathway in the maintenance of the active NSC subpopulation (Nyfeler et al., 2005; Androutsellis-Theotokis et al., 2006; Breunig et al., 2007; Ables et al., 2010; Aguirre et al., 2010; Basak et al., 2012). In contrast, activation of the RBP-J mediated canonical Notch pathway, potentially promoted by Dll1 ligand expressed by proliferating NSCs and IPs, preserves the quiescent NSC pool (Carlen et al., 2009; Ehm et al., 2010; Imayoshi et al., 2010; Basak et al., 2012; Kawaguchi et al., 2013). These data suggest that Notch can differentially regulate active and quiescent NSC populations in a context-dependent manner. Given that *Hes5* is expressed in both dividing and dormant adult NSCs, Notch is unlikely to be the only key to NSC heterogeneity (Lugert et al., 2010; Giachino et al., 2014). However, differences in the strength, dynamics, and targets of the pathway may contribute to heterogeneity and explain the discrepancies observed in mouse mutant phenotypes. Low levels of Notch activation (NICD) for instance promote proliferation of embryonic neural progenitors, whereas high levels lead to growth arrest *in vitro* (Guentchev and McKay, 2006). Therefore, one possibility is that the differential actions of Notch on proliferation vs. quiescence of adult NSCs are a function of dose. Another possibility is that oscillatory vs. sustained expression of Notch targets differentiate active and quiescent NSC pools. In mouse embryonic NSCs and progenitors, levels of Hes1/Hes5 oscillate in anti-phase with their repressed target Ascl1, and this oscillatory expression promotes proliferation (Imayoshi et al., 2013). In contrast, sustained Hes1 expression inhibits NSC proliferation and neurogenesis in the developing central nervous system (Baek et al., 2006). It is currently not known if the expression of *Hes* genes is sustained or oscillatory in adult NSCs, and the available tools are not adequate to address this issue *in vivo*. However, oscillatory expression of Notch targets together with low refractory expression of Ascl1 in adult NSCs that are in the cell-cycle would be compatible with data showing that the *Ascl1::CreER* locus can be used to lineage trace a subpopulation of neurogenic NSCs *in vivo* (Kim et al., 2011). Conversely, quiescent adult NSCs express high levels of Id proteins (Nam and Benezra, 2009), which can interfere with the negative autoregulation of *Hes1* and therefore modulate its oscillatory expression (Bai et al., 2007). These findings prompt to speculate that *Hes* genes are persistently expressed at high levels in quiescent NSCs.

Another possible scenario to explain the multifaceted functions of Notch in adult NSCs would be that distinct pathway components are differentially engaged in Notch signaling activity in different cell subpopulations. It has been proposed that Notch1 is required to maintain the active adult NSC pool but is dispensable during quiescence (Ables et al., 2010; Basak et al., 2012). This suggests that other members of the Notch family could provide a maintenance signal for quiescent NSCs and compensate for the absence of Notch1 in this population (Basak et al., 2012). Heterogeneity of Notch activity is related to cellular diversity in the neurogenic niches of the adult zebrafish brain, where Notch3 gates NSC activation whereas Notch1b maintains activated progenitors (Alunni et al., 2013). Interestingly, Notch3 restricts stem cell activation in muscle and mammary, while Notch1 is associated with proliferation (Carlson et al., 2008; Kitamoto and Hanaoka, 2010; Bjornson et al., 2012; Mourikis et al., 2012; Lafkas et al., 2013). It is tempting to speculate that distinct Notch receptors differentially regulate quiescent and active stem cell subpopulations in the adult mammalian brain. In addition to NSC heterogeneity within the same lineage, independent NSC lineages are fated to generate distinct OB neurons in the SVZ. Lineage tracing experiments with *Hes5::CreER* transgenic mice demonstrated that NSCs with active canonical Notch signaling generate multiple neuron subtypes in the OB (Giachino et al., 2014) implying that all adult NSC lineages require Notch for maintenance. However, this does not exclude that different receptors or receptor combinations mediate Notch activity within the NSCs of each lineage. Interestingly, genetic fate mapping of cells expressing individual Notchs revealed multiple lineages in bone marrow and mammary gland (Lafkas et al., 2013; Oh et al., 2013; Sale et al., 2013).

Not only the activity of Notch paralogues, but also combinations of different Notch target genes in NSC subpopulations may be a source of heterogeneity. NSCs in the developing neural tube of the mouse are heterogeneous based on their expression of *Hes* family genes, with cohorts of radial glia cells expressing either *Hes1* or *Hes5*, or both (Basak and Taylor, 2007; Nelson et al., 2013). Brain lipid binding protein (BLBP), a direct target of Notch signaling (Anthony et al., 2005), is also expressed heterogeneously by radial glia (Hartfuss et al., 2001). Recently, it was shown that different combinations of *Her/Hes* family genes correlate with the proliferation rate of NSCs and progenitors in the adult zebrafish (Chapouton et al., 2011). In the same line, BLBP expression positively correlates with proliferation of *Hes5*-expressing NSCs in the adult mouse SVZ, whereas most quiescent NSCs express *Hes5* but not BLBP (Giachino et al., 2014). How combinatorial or selective expression of Notch targets regulates the fate and activity of adult NSC subpopulations deserves closer scrutiny in the future.

## Implications of adult NSC heterogeneity for aging and regeneration

The adult mammalian neurogenic niches show a remarkable capacity for self-repair and remodeling in response to lesion (Doetsch et al., 1999; Seri et al., 2001; Kuo et al., 2006; Nomura et al., 2010). However, neurogenesis declines with age in both the SVZ and SGZ (Lazarov et al., 2010) and most neuron subtypes produced in the OB are equally affected (Shook et al., 2012). Understanding the basis of this overall age-related decline in neurogenesis, and the mechanisms controlling self-renewal over the life on an individual, is fundamental to exploit the regenerative potential of adult NSCs.

One cause of reduced neurogenesis during aging is the impairment of the NSC compartment. An open question is whether this impairment reflects depletion or increased quiescence of NSCs (Hattiangady and Shetty, 2008; Lugert et al., 2010; Encinas et al., 2011; Shook et al., 2012; Giachino et al., 2014). This issue remains debated as most studies did not take into account the differential effects that aging may have on NSC subpopulations. One possibility is that active NSCs are more susceptible to imbalances between maintenance and differentiation signals that may occur during aging. These imbalances affect self-renewal, and therefore active NSCs are preferentially lost compared to quiescent populations (Lugert et al., 2010; Encinas et al., 2011; Giachino et al., 2014). Furthermore, some quiescent NSCs may activate to replenish an exhausted active NSC pool and thereby also be lost during aging. This is supported by findings that quiescent NSCs are reduced with time following depletion of the active NSC pool either by genetic means (Basak et al., 2012), or chronic treatment with antimitotic drugs (Doetsch et al., 1999). However, a variable but substantial proportion of NSCs is preserved in both the SGZ and SVZ of old mice, but these are dormant rather than senescent and can be reactivated (Jin et al., 2003; Hattiangady and Shetty, 2008; Lugert et al., 2010; Giachino et al., 2014). It will be important to genetically trace the progeny of distinct NSC populations during aging to assess differences in their lifespan, lineage plasticity and regenerative potential. Understanding the mechanisms responsible for NSC dormancy in the mouse neurogenic niches could help to understand why most NSCs in the human brain stop generating neurons after birth (Sanai et al., 2011).

### Conflict of interest statement

The authors declare that the research was conducted in the absence of any commercial or financial relationships that could be construed as a potential conflict of interest.
